# Robotic Distal Pancreatectomy Yields Superior Outcomes Compared to Laparoscopic Technique: A Single Surgeon Experience of 123 Consecutive Cases

**DOI:** 10.3390/cancers15225492

**Published:** 2023-11-20

**Authors:** Hao Ding, Michal Kawka, Tamara M. H. Gall, Chris Wadsworth, Nagy Habib, David Nicol, David Cunningham, Long R. Jiao

**Affiliations:** 1 Department of Surgery and Cancer, Imperial College London, Exhibition Road, South Kensington, London SW7 2BU, UK; hao.ding19@imperial.ac.uk (H.D.); michal.kawka17@imperial.ac.uk (M.K.); tamara.gall1@nhs.net (T.M.H.G.); 2 Department of Surgery and Oncology, The Royal Marsden Hospital, 203 Fulham Road, London SW3 6JJ, UK; david.nicol@rmh.nhs.uk (D.N.); david.cunningham@rmh.nhs.uk (D.C.); 3 Department of Gastroenterology and Surgery, The Hammersmith Hospital, Du Cane Road, London W12 0HS, UK; christopher.wadsworth@nhs.net (C.W.); nagy.habib@imperial.ac.uk (N.H.)

**Keywords:** distal pancreatectomy, robotic, laparoscopic, minimally invasive surgery, pancreatic cancer

## Abstract

**Simple Summary:**

Pancreatic tumours usually require surgical removal. With advancing technology, these operations can be performed using laparoscopic and robotic techniques, which reduce surgical trauma to patients compared to conventional open surgery. In theory, robotic surgery should yield better outcomes due to its superior precision and control. Our study provided more evidence in support of this theory after comparing 62 patients who had laparoscopic surgery with 61 patients who had robotic surgery for the removal of their pancreatic tumours. We found that robotic surgeries resulted in fewer cases of conversion to open operations and fewer complications.

**Abstract:**

Technical limitations of laparoscopic distal pancreatectomy (LDP), in comparison to robotic distal pancreatectomy (RDP), may translate to high conversion rates and morbidity. LDP and RDP procedures performed between December 2008 and January 2023 in our tertiary referral hepatobiliary and pancreatic centres were analysed and compared with regard to short-term outcomes. A total of 62 consecutive LDP cases and 61 RDP cases were performed. There was more conversion to open surgeries in the laparoscopic group compared with the robotic group (21.0% vs. 1.6%, *p* = 0.001). The LDP group also had a higher rate of postoperative complications (43.5% vs. 23.0%, *p* = 0.005). However, there was no significant difference between the two groups in terms of major complication or pancreatic fistular after operations (*p* = 0.20 and *p* = 0.71, respectively). For planned spleen-preserving operations, the RDP group had a shorter mean operative time (147 min vs. 194 min, *p* = 0.015) and a reduced total length of hospital stay compared with the LDP group (4 days vs. 7 days, *p* = 0.0002). The failure rate for spleen preservation was 0% in RDP and 20% (n = 5/25) in the LDP group (*p* = 0.009). RDP offered a better method for splenic preservation with Kimura’s technique compared with LDP to avoid the risk of splenic infarction and gastric varices related to ligation and division of splenic pedicles. RDP should be the standard operation for the resection of pancreatic tumours at the body and tail of the pancreas without involving the celiac axis or common hepatic artery.

## 1. Introduction

Despite advances in technology and operative techniques, open distal pancreatectomy (ODP) is still considered the gold standard in many centres worldwide. It is associated with morbidity rates ranging from 22% to 47%, with postoperative pancreatic fistula (POPF) as the most common complication [1,2,3,4,5]. Minimally invasive distal pancreatectomy (MIDP) with both laparoscopic and robotic platforms has shown consistently comparable oncological and survival outcomes to ODP [6,7,8,9]. Moreover, studies have demonstrated clear benefits of MIDP with respect to short and medium-term outcomes [7,8,9]. A large multicentre trial has shown that minimally invasive resections of left-sided pancreatic tumours were associated with longer operative times but lower operative blood loss compared to ODP [7]. MIDP, particularly robotic surgery, may also result in a low R1 resection rate for pancreatic ductal adenocarcinoma (PDAC) with its enhanced views and improved precision [8]. A pan-European retrospective score-matched study involving 1212 patients with PDAC confirmed short-term clinical advantages of MIDP, including less intraoperative blood loss and shorter postoperative hospital stay [9] but a high rate of conversion to OPD [10]. 

Although MIDP has evolved over the past decade [11,12], consensus regarding the outcomes of the robotic compared with the laparoscopic approach to distal pancreatectomy is limited. Several meta-analyses have suggested comparable surgical outcomes between the two approaches and lower conversion rates for robotic distal pancreatectomy (RDP). However, drawbacks of RDP, such as higher cost and long learning curve and operative time using a robotic platform, may need to be taken into consideration [13,14,15]. Since the introduction of RDP in 2001 [13], there has been a slow introduction of robotic pancreatic surgery worldwide, and many surgeons and centres remain on their initial learning curve. Proficiency in RDP has been shown to be achieved after completion of 10–40 cases, suggesting a shortened learning curve compared to laparoscopic surgery [14] supported by our own randomised control trial (RCT) on basic surgical skills learning comparing robotic with laparoscopic training [15].

Existing limitations of laparoscopic distal pancreatectomy (LDP) include two-dimensional imaging with resultant limited depth perception and ergonomics, the fulcrum effect, and the augmented tremor of effect or instruments [16]. Robotic surgery can theoretically overcome some of the technical disadvantages of the laparoscopic approach by providing a three-dimensional, high-definition surgical view and a larger range of motion [17,18]. Ligation and division of small vessels may be facilitated with RDP, resulting in higher rates of spleen preservation. There may also be less risk of conversion to open surgery and a shorter length of hospital stay [19]. However, more evidence is needed to reach a conclusion on whether RDP is superior to LDP [20]. Although a properly run RCT would be ideal to address some of the issues related to this by providing the level 1 evidence, our view is that, practically, it will be extremely difficult to conduct such a trial without inferior both patients’ and surgeons’ choice and introducing bias. Furthermore, almost all surgeons would switch from a laparoscopic or open technique to the robotic platform once the robotic skill is acquired. The aim of this study is to compare the short-term surgical outcomes and the success rate of spleen preservation with the intention to treat all RDP and LDP procedures performed in tertiary referral hepatobiliary and pancreatic centres since the introduction of laparoscopic pancreatic surgery in 2008 and robotic pancreatic surgery in 2017. 

## 2. Materials and Methods

### 2.1. Study Population

This was a retrospective review of a prospectively maintained database of all consecutive patients who underwent laparoscopic distal pancreatectomy (LDP) from December 2008 (date of first LPD case) to December 2018 and those who underwent robotic distal pancreatectomy (RDP) from January 2017 (date of first RPD case) to January 2023. All procedures were performed in West London tertiary referral Hepatobiliary and Pancreatic centres at Hammersmith and the Royal Marsden Hospitals. The senior surgeon (LRJ), who has performed 123 ODP before transitioning to LPD, also performed over 100 cases of both laparoscopic and robotic pancreaticoduodenectomy [21]. Currently, the centre volume at the Royal Marsden Hospital, where all the RDP is performed, is 180 major pancreatic resections annually consisting of pancreaticoduodenectomy (n = 140, 77.8%) and distal pancreatectomy (n = 40, 22.2%), shared between two senior surgeons.

All pancreatic resections were performed minimally invasively unless the tumour involved major vascular pedicles. For MIDP, patients with tumours involving the celiac axis and/or common hepatic artery were excluded. For pancreatic cancer at the body and tail of the pancreas, patients with borderline resectable tumours involving major vascular pedicles such as the coeliac axis and/or common hepatic artery and/or surrounding structures such as stomach and colon were offered upfront systemic chemotherapy routinely. Since January 2017, both robotic and laparoscopic DP were performed until December 2018, when a complete transition to robotic DP was undertaken. Only patients with a benign pancreatic tumour were considered for spleen-preserving distal pancreatectomy. 

### 2.2. Outcomes

Clinicopathological data, including age, sex, surgical approach (laparoscopic/robotic), conversion to open approach, operative time, estimated blood loss (EBL), and peri-operative transfusion rate, were extracted. Pathologic data, including preoperative histology and postoperative histopathologic diagnosis, were recorded. In patients who had malignant tumour resection status, tumour size, T stage, perineural invasion, vascular invasion, and number of positive lymph nodes were also collected. Postoperative morbidity and mortality within 90 days after surgery were recorded. Morbidity was graded using the Clavien-Dindo Classification [22]. The length of total hospital stay was recorded, as well as the length of follow-up and disease recurrence after surgery. POPF is analysed with the International Study Group (ISGPS) definition and grading [23]. 

### 2.3. Statistical Analysis 

All statistical analyses were conducted using the computer programme Statistical Package for Social Sciences for Windows, version 27.0 (SPSS Inc, Chicago, IL, USA). Continuous variables were expressed as mean ± standard deviation or median and range. Continuous variables were compared using Student’s *t*-test (for normally distributed data) or Mann–Whitney U test (for nonparametric data). Categorical variables were compared using χ^2^ test (when the sample size of all groups is >5) or Fisher’s exact test (when the sample size of one group is <5). All tests were two-sided, and *p* < 0.05 was considered statistically significant. This study was conducted in line with the declaration of Helsinki and was conducted and registered as a clinical audit, as no deviations from standard care were made, and all data were routinely collected.

## 3. Results

### 3.1. Demographic Details

A total of 123 patients underwent LPD (n = 62, 50.4%) and RPD (n = 61, 49.6%) during the study period. The laparoscopic and robotic groups were comparable with respect to baseline characteristics (Table 1).

### 3.2. Histopathology Details

The pathologic diagnoses of resected lesions are shown in Table 1. The most common indication for distal pancreatectomy (DP) was neuroendocrine tumour (n = 26, 21.1%). The most common indication for surgery in the LDP cohort was also neuroendocrine tumour (n = 17, 27.4%), followed by pancreatic ductal adenocarcinoma (PDAC, n = 12, 19.4%), while the leading indication for RDP was mucinous cystic neoplasm (n = 16, 26.2%) followed by PDAC (n = 13, 21.3%). Overall histology of the two groups is comparable (*p* = 0.283). Malignant tumour characteristics such as tumour size, T stage, and resection status showed no significant differences between the two groups (Table 1).

### 3.3. Operative and Postoperative Details 

The conversion to open resection rate was significantly higher in the laparoscopic group (21.0% vs. 1.6%, *p* = 0.001) (Table 1). The LDP group also had higher morbidity (43.5% vs. 23.0%, *p* = 0.005) with seven major complications compared to three in the RDP group. Seven patients developed POPF in the entire cohort, consisting of four in the LDP group and three in the RDP group. Three POPF cases in the LPD group required radiological drainage, and all the rest were managed conservatively. Both the operative time and the number of operations with more than 500 mL of estimated blood loss were comparable between the two groups. For non-malignant tumours, the number of patients who underwent DP with splenectomy was not significantly different between the two groups (*p* = 0.441). No mortality was observed in either group within 90 days of surgery. 

For spleen-preserving operations, the operative time was significantly shorter in the RDP group compared with LDP (194 ± 89.4 min vs. 147 ± 45.3 min, *p* = 0.015) (Table 2). The length of hospital stay was shorter after robotic spleen-preserving operations (4 days vs. 7 days, *p* = 0.0002). The surgical technique also differed significantly between the two groups, with 18 out of 25 cases of LDP using the Warshaw technique (WT) with the division of the splenic vascular pedicles distal to the splenic hilum and 27 out of 31 cases of RDP using the Kimura technique (KT) with the preservation of the splenic vascular pedicles. With the intention to treat spleen preservation, the failure rate for spleen-preserving operation was significantly diminished robotically when compared with that laparoscopically (n = 0, 0% vs. n = 5, 20%, *p* = 0.009). Five cases of laparoscopic spleen-preserving DP were converted to open DP with splenectomy because of intraoperative bleeding from splenic vein (n = 2, 40%) and technical difficulties from failure to dissect the tumour off splenic vein safely with a combination of laparoscopic instruments (n = 3, 60%).

## 4. Discussion

This study is a direct comparison between RDP and LPD since their respective introduction in a tertiary referral HPB unit; it is the largest series of both laparoscopic and robotic DP performed and reported in the UK where centralisation of hepatobiliary and pancreas service occurred over two decades ago to serve minimally two million population. Our results provide further evidence that RDP is safe and feasible without increased morbidity and mortality compared with LDP. Furthermore, the rate of conversion to open surgery is significantly low in the RDP group, with a diminished failure rate for spleen preservation.

The low conversion rate for RDP has previously been reported in several other case series [24,25,26] and is confirmed in a recently published meta-analysis [27]. Reasons for conversion to open surgery may include vascular involvement, bleeding, poor visualisation, difficulty in distal pancreas dissection, extended resection, and concern for oncological radicality. Conversion from MIDP to open surgery is associated with increased morbidity, serious morbidity, and organ space infection [28]. RDP’s low conversion rate might be due to its increased dexterity capacities and precision as well as 3D vision, which enables improved accuracy when operating on small vascular branches. The rate of conversion to open surgery in the RDP group was significantly lower in our study. This might also contribute to the senior surgeon’s prior laparoscopic experience in pancreatic resection before starting robotic surgery. For spleen-preserving distal pancreatectomy, we found a significantly diminished rate of unplanned splenectomy rate with a 100% successful spleen preservation in the RDP group compared with LDP, which is consistent with previous reports [19,20]. 

A systematic review of 32 studies suggested that there were no significant differences between the learning curves for LDP and RDP [28]. However, in most published data and our series, we believe that the prior experience of laparoscopic surgery is valuable in robotic surgery [29]. In our series, robotic surgery was introduced only after proficiency had already been gained in laparoscopic surgery by the senior surgeon. The operative time was longer for LDP compared with RDP, although this did not reach statistical significance (177 min vs. 150 min, *p* = 0.054). The superiority of either technique regarding operative time is therefore not clear. Published studies have shown contradictory results [24,30], and Di Martino et al.’s meta-analysis concluded that there was no significant difference between operative time for RDP and LDP [31]. No significant difference in the overall POPF rate (n = 4, 6.5% vs. n = 3, 4.9%, *p* = 0.713) was seen among the two cohorts, as confirmed by a previous comparative meta-analysis [13,20]. The overall complication rate was significantly higher for both minor and major complications, and there was a longer length of stay in the LPD group compared with the RDP group. However, LDP and RDP were performed at two different hospitals. While all the LDPs were performed at the Hammersmith Hospital, the RDPs were all carried out at the Royal Marsden Hospital over different periods. Hence, the difference in standard of care may have had an impact on the length of stay. 

Although technically challenging to perform, minimally invasive spleen-preserving distal pancreatectomy should be considered the gold standard operation for patients with benign pancreatic conditions to prevent its related postoperative complications and lifelong risks of post-splenectomy sepsis syndrome with a mortality rate of up to 50% [32]. Two surgical techniques have been described for spleen-preserving distal pancreatectomy. The WT with ligation and division of splenic vascular pedicles distal to splenic hilum relies on splenic blood supply entirely from the short gastric and left gastroepiploic vessels. The KT preserves the splenic vascular pedicles by meticulous dissection and protects the splenic vessels during distal pancreatectomy. WT is less technically demanding, faster, and easier than KT. However, WT carries significantly higher risks of infarction of the spleen, requiring further operation and long-term risks of varices around the splenic hilum [33]. Technically, with the intention to treat for spleen preservation, none of the robotic spleen-preserving distal pancreatectomies in our series failed to complete because of intraoperative difficulties encountered during the dissection of splenic vessels. In the LDP group, five cases were converted to open and underwent open distal pancreatectomy and splenectomy due to intraoperative bleeding in two cases and difficulty in completing the operation safely in three cases. Furthermore, due to the technical advantages of the Da Vinci robotic vision and instrumentation over laparoscopic surgery, more patients in our group underwent spleen-preserving distal pancreatectomy with KT—a better method for preservation of splenic vascular pedicles and spleen. POPF rate did not differ between the techniques. However, all three cases of PDPF required radiological drainage in the LDP group. 

To our knowledge, this is the only series in the UK and one of the few large series in the West on LDP and RDP that directly compares LDP with RDP for spleen preservation. We believe that patients with non-malignant conditions in the body and tail of the pancreas should be offered spleen-preserving distal pancreatectomy as the gold standard. Our results clearly showed the benefits and advantages of RDP for spleen preservation in terms of the success rate of spleen preservation, intraoperative outcomes, postoperative complications, and length of hospital stays. However, for tumours located close to the splenic hilum and splenic vessels embedded in the pancreas, spleen preservation may not be technically possible. Some biases cannot be excluded in retrospective studies of this study. 

The main limitation of this study is its retrospective nature and the inherent selection bias. However, the selection bias was reduced as the same surgeon performed all the laparoscopic and robotic operations over the same transition period of the learning phase and proficiency phase. Patients involved fulfilled eligibility for both minimally invasive approaches with the same inclusion and exclusion criteria for LDP and RDP with or without splenectomy. We believe this is the best objective evidence on robotic and laparoscopic surgery without introducing selection and surgeon’s bias, given the difficulties related to RCTs comparing laparoscopic with robotic pancreatic surgery. Although the learning curve effect might skew the data for the first few RDP cases included, this would also apply to the first few LPD cases by the same surgeon. Having said that, as minimally invasive surgery skills exist on the continuum, the previous LPD experience might have influenced the proficiency in RPD, affecting the results. As such, randomised studies aimed at eliminating selection bias, with pre-specified proficiency thresholds for participating centres and surgeons, should be conducted in the future to better delineate the true benefit RPD might have over LPD. Further analyses should also include information about health-related quality of life, which is emerging as an important endpoint in research studies and audits to ensure that technical improvements translate to patient satisfaction and long-term survival outcomes in PDAC patients. We will be reporting this once an adequate number of PDAC patients is reached.

Robotic surgical techniques have been increasingly adopted for distal pancreatectomy across the globe over the past decade. It is still not conclusive as to whether it holds a clear advantage in comparison to the laparoscopic approach in those already trained in advanced laparoscopic skills. However, we expect that a robotic platform will enable more pancreatic surgeons to perform MIDP rather than open surgery, as developing advanced skills is easier with a robotic compared to a laparoscopic technique [18]. RDP should be the gold standard surgery for distal pancreatectomy with or without spleen preservation in patients without celiac axis or common hepatic artery involvement. With better access to both robotic training and robotic theatres over the next 5–10 years and a likely cost reduction with the launch of other robotic surgical companies, we predict that the number of robotic pancreatic resections performed will increase significantly in high-income countries. 

## 5. Conclusions

In our study, RDP reduced the conversion rate when compared with LDP and decreased the postoperative complication rate, leading to a shorter length of stay. Also, RDP was superior to LPD for spleen preservation. More robust evidence is needed to validate these findings, ideally coming from large, multicentre, prospective studies. 

## Figures and Tables

**Table 1:** Demographics, Pathological Indications, and Perioperative Outcomes for MIDP.

Variable	Total DP (n = 123)	Laparoscopic DP (n = 62)	Robotic DP (n = 61)	*p* Value
**Demographics**				
Sex (Male: Female, n)	51: 72	26: 36	25: 36	0.92
Age (median, years, (range))	63 (25–86)	62.5 (29–85)	64 (25–86)	0.81
**Histopathology (n, (%))**				0.28
***Benign pancreatic disease***	**11 (8.9)**	**7 (11.3)**	**4 (6.6)**	**-**
Pancreatic pseudocyst	4	3	1	**-**
Chronic pancreatitis	7	4	3	**-**
***Benign pancreatic tumour***	**52 (42.3)**	**21 (33.9)**	**31 (50.8)**	**-**
Serous cystic neoplasm	4	4	0	**-**
Intraductal papillary mucinous neoplasm	22	11	11	**-**
Mucinous cystic neoplasm	21	5	16	**-**
Solid pseudopapillary tumour	5	1	4	**-**
***Malignant pancreatic tumour***	**51 (41.5)**	**29 (46.8)**	**22 (36.1)**	**-**
Neuroendocrine tumour	26	17	9	**-**
Pancreatic ductal adenocarcinoma	25	12	13	**-**
Others *	**9 (7.3)**	**5 (8.1)**	**4 (6.6)**	**-**
Histology of malignant tumours				
Tumour size (mean ± SD, mm)	37.5 ± 23.2	32.6 ± 23.2	43.7 ± 23.0	0.11
***T Stage (n)***				**0.28**
1	14	7	7	-
2	17	8	9	-
3	16	12	4	-
4	4	3	1	-
***Resection Status (n)***				**0.14**
R0	43	22	21	-
R1	7	6	1	-
R2	1	1	0	-
Number of lymph nodes harvested (median, (range))	16 (0-43)	18 (0-43)	13 (2-27)	0.65
**Intraoperative outcomes**				
Operative time (mean ± SD, mins)	159 ± 62.9	177 ± 76.4	150 ± 52.9	0.05
Estimated blood loss > 500 mL (n, (%))	4 (3.3)	3 (4.8)	1 (1.6)	0.66
Splenectomy in non-malignant cases (n, (%))	17 (13.8)	7 (11.3)	10 (16.4)	0.44
Conversion to open (n, (%))	14 (11.4)	13 (21.0)	1 (1.6) *	0.001
**Postoperative outcomes**				
90 Day Mortality (n)	0	0	0	-
***Complications (n, (%))***	41 (33.3)	27 (43.5)	14 (23.0) *	0.005
Minor complications of CD grade 1–2	31	20	11	0.07
Major complications of CD grade 3–5	10	7	3	0.20
Pancreatic fistula (n, (%))	7 (5.7)	4 (6.5)	3 (4.9)	0.71
**Length of total hospital stay (median, days, (range))**	6 (4–33)	7 (4–33)	6 (6–14) *	0.0008

* Other histology included focal hypertrophy (n = 1), desmoid fibromatosis (n = 1), periductal fibrosis (n = 1), pancreatic intraepithelial neoplasia (n = 1), and pancreatic gastrointestinal stromal tumour (n = 1) in the LDP group; pancreatic intraepithelial neoplasia (n = 1), splenunculus (n = 1), and metastatic renal cancer (n = 2) in the RDP group.

**Table 2:** Operative Details and Outcomes of MIDP with Spleen Preservation.

Perioperative Outcomes	Laparoscopic DP (n = 25)	Robotic DP (n = 31)	*p* Value
**Intraoperative outcomes**			
Operative time (mean ± SD, mins)	194.0 ± 89.4	147.0 ± 45.3	0.015
Estimated blood loss > 500 mL (number of cases)	0	0	--
Convert to open (number of cases)	5	0	0.009
Splenectomy (number of cases)	5	0	0.009
**Surgical techniques (number of cases)**			**<0.001**
Warshaw	18	4	
Kimura	7	27	
**Histology**			
***Number of lymph nodes harvested (median, (range))***	2 (26.0–0)	4 (12.0–2.0)	0.211
Tumour size (mm, mean ± SD)	29.0 ± 9.3	40.3 ± 21.7	0.021
Postoperative outcomes			
**Complications (number of cases)**			0.670
Minor complications of grade 1–2	9	5	
Major complications of grade 3–5	2	2	
Pancreatic fistula	3	3	0.99
**Radiological drainage (number of cases)**	3	0	0.047
Length of total hospital stays (median, days, (range))	7 (5.0–20.0)	4 (3.0–13.0)	<0.001

## Data Availability

Data are unavailable due to privacy or ethical restrictions.
